# Going, Toll-like receptors in skin inflammation and inflammatory diseases

**DOI:** 10.17179/excli2020-3114

**Published:** 2021-01-07

**Authors:** Vijay Kumar

**Affiliations:** 1 Children Health Clinical Unit, Faculty of Medicine and Biomedical Sciences, Mater Research, University of Queensland, ST Lucia, Brisbane, Queensland 4078, Australia; 2 School of Biomedical Sciences, Faculty of Medicine, University of Queensland, ST Lucia, Brisbane, Queensland 4078, Australia

**Keywords:** skin, TLRs, inflammation, psoriasis, atopic dermatitis, melanoma

## Abstract

The Indian Ayurvedic physicians knew the concept of inflammation dating back to 1500 BC. The continuous progress in the immunology of inflammation has explained its undiscovered mechanisms. For example, the discovery of Toll-like receptor 4 (TLR4) in humans (1997) has revolutionized the field of infection biology and innate immunity. The laboratory mice have shown twelve TLRs and express TLR10 (CD290) as a disrupted pseudogene, and humans have ten functional TLRs. Now, it is well established that TLRs play a significant role in different infectious and inflammatory diseases. Skin inflammation and other associated inflammatory diseases, including atopic dermatitis (AD), acne vulgaris, and psoriasis, along with many skin cancers are major health problems all over the world. The continuous development in the immunopathogenesis of inflammatory skin diseases has opened the window of opportunity for TLRs in studying their role. Hence, the manuscript explores the role of different TLRs in the pathogenesis of skin inflammation and associated inflammatory diseases. The article starts with the concept of inflammation, its origin, and the impact of TLRs discovery on infection and inflammation biology. The subsequent section describes the burden of skin-associated inflammatory diseases worldwide and the effect of the geographical habitat of people affecting it. The third section explains skin as an immune organ and explains the expression of different TLRs on different skin cells, including keratinocytes, Langerhans cells (LCs), skin fibroblasts, and melanocytes. The fourth section describes the impact of TLRs on these cells in different skin-inflammatory conditions, including acne vulgaris, AD, psoriasis, and skin cancers. The article also discusses the use of different TLR-based therapeutic approaches as specific to these inflammatory skin diseases.

## Introduction

Inflammation was known to ancient physicians dating back to 1500 BC and 600 AD practicing Ayurvedic medicine in the Indian peninsula under different names in different contexts, namely Shotha and Shopha (Kumar, 2019[100]; Rao, 2008[162]). The process of inflammation and the edema associated with inflammatory damage both got attention due to the descriptions in Ayurveda as pathological manifestations of a disease. An elevation in the affected area, edema, heaviness, and pain are the morphological manifestations of inflammation. Later on, the ancient Egyptian and Greek cultures described inflammation, for example, Hippocrates in the 5^th^ century BC described the *edema*. Aulus Celsus (30 BC-38 AD) described the inflammation by its four cardinal/fundamental signs called *rubor* (redness), *calor* (increased heat), *tumor* (swelling), and *dolor* (pain) (Rocha e Silva, 1994[163]). This definition of inflammation is more frequently used to recognize classical acute inflammation occurring due to profound innate immune response in acute trauma or infection. Galen, the physician, and surgeon of Roman emperor Marcus Aurelius has been often credited with introducing the fifth sign of inflammation that is the loss of function in the affected tissue/organ. The humoral view of inflammation (and pus formation specifically), introduced by Galen, regarded inflammation as beneficial to the host and not just a superimposed pathology (Rocha e Silva, 1994[163]). However, the definitive description of the fifth sign of inflammation called *function laesa* (loss of function) was added by *Virchow* in 1871 and viewed inflammation as inherently pathological to the host (Ley, 2001[112]; Scott et al., 2004[183]). In modern medicine, the term inflammation has originated from the Latin word *inflammare* (to set on fire) (Scott et al., 2004[183]). Today, inflammation is recognized as a healing response of the body in acute stages of tissue injury, for example, in response to cell injury due to trauma or infection, wherein a complex network of molecular and cellular interactions direct the restoration of homeostasis and tissue repair (Fullerton and Gilroy, 2016[56]; Ortega-Gómez et al., 2013[149]; Serhan et al., 2007[186]). However, severe and systemic acute inflammation may result in pathology, organ failure, and death as it happens in sepsis. The persistence of inflammation for a longer duration (months and years) may often lead to different chronic inflammatory diseases, including auto-inflammatory diseases, autoimmune diseases, and various cancers such as colorectal cancer, breast cancer, lung cancer, and prostate cancer (Coussens and Werb, 2002[27]; Rakoff-Nahoum, 2006[160]). 

The discovery of Toll-like receptors (TLRs) proved crucial in understanding the pathogenesis of inflammation (Kumar, 2018[101]). TLRs constitute a group of so-called pattern recognition receptors (PRRs) as they recognize many microbe or pathogen-associated molecular patterns (MAMPs or PAMPs) and molecular patterns originated from stressed and dying cells called damage or death-associated molecular patterns (DAMPs) or alarmins (Garg and Agostinis, 2017[58]; Yang et al., 2017[224]). Different immune and non-immune cells express TLRs, and the author has discussed their detail somewhere else (Kumar, 2018[101]). In addition to TLRs, several types of other PRRs have also been discovered that also recognize different PAMPs and DAMPs. They include nod-like receptors (NLR), RIG-like helicases (RLH), C-type lectin-like receptors (CLRs), Triggering receptors expressed on myeloid cells (TREM-1), and more recently described DNA sensors, cyclic GMP-AMP synthase (cGAS) and gamma-interferon-inducible protein Ifi-16 (IFI-16) or p204 (a PYHIN (pyrin and HIN200 domain-containing protein) protein that serves as an intracellular DNA sensor to mediate type 1 interferon or IFN production) (Almine et al., 2017[7]; Brown et al., 2018[16]; Kawai and Akira, 2009[93]; Kumar, 2019[99]; Roe et al., 2014[164]; Unterholzner et al., 2010[212]). By far, TLRs are the most studied PRRs in the context of immunity, inflammation, and inflammatory diseases, including cancer, sepsis, acute lung injury/inflammation (ALI), neuroinflammation, discussed somewhere else (Huang et al., 2018[72]; Kumar, 2018[101]; Kumar, 2019[103]; Kumar, 2020[102]; Rakoff-Nahoum and Medzhitov, 2009[161]). The current review discusses the role of TLRs in skin inflammation and inflammatory diseases.

## Incidence and Impact of Skin Inflammation and Inflammatory Diseases

Skin inflammation plays a crucial role in different skin infections, including leishmaniasis, *Staphylococcus aureus*-induced skin infection, acne vulgaris, and sterile inflammatory diseases, including atopic dermatitis (AD), psoriasis, and different skin cancers, including melanoma. Skin diseases are the 4^th ^leading cause of non-fatal disease burden worldwide according to the Global Burden of Disease project (Seth et al., 2017[187]). However, skin diseases exert financial and social impact on the patient's life. For example, in 2013, the annual cost related to psoriasis summed to $112 billion in the USA only that may have further increased in 2020 (Brezinski et al., 2015[15]). Also, AD in 2015 in the USA alone cost $5.297 billion, and people with AD change their occupation (Drucker et al., 2017[38]). The skin cancer called melanoma management in different European countries, including France, Germany, and United Kingdom (UK) costs very high that are €13.1 million (France), €30.2 million (Germany), and €27.8 or £22.2 million (UK) annually (Grange et al., 2017[62]). The changing lifestyles and the environment that impact host genes and immunity comprise crucial factors for the increasing incidence of inflammatory skin diseases (Griffiths et al., 2017[63]; Kantor and Silverberg, 2017[90]; Martin et al., 2020[125]; Solomon et al., 2019[195]). Vitamin D also plays a crucial role in skin diseases and immunity. The exposure to sunlight (ultraviolet or UV rays) induces vitamin D production in the epidermis through the photolysis of provitamin D3 to previtamin D3 (Holick et al., 1987[69]). The previtamin D3 may either isomerize to vitamin D3 (Cholecalciferol) or photolyze to lymisterol and tachystreol. However, vitamin D is also sensitive to the sunlight and photolyzes to 5,6-transvitamin D3, suprasterol I, and suprasterol II (Holick et al., 1987[69]). The active vitamin D (3)-1,25-dihydroxyvitamin D(3) (1,25D3) also increases the TLR2 expression in the skin (Liu et al., 2006[117]). Thus, the population residing in countries with low exposure to sunlight are more prone to have certain autoimmune diseases and skin infections, and inflammatory diseases. Hence, it becomes crucial to identify novel immune pathways important for their pathogenesis to identify novel target-based therapeutics. 

## Skin as an Immune Organ and TLR Expression in Different Skin Cells

**Figure 1:**
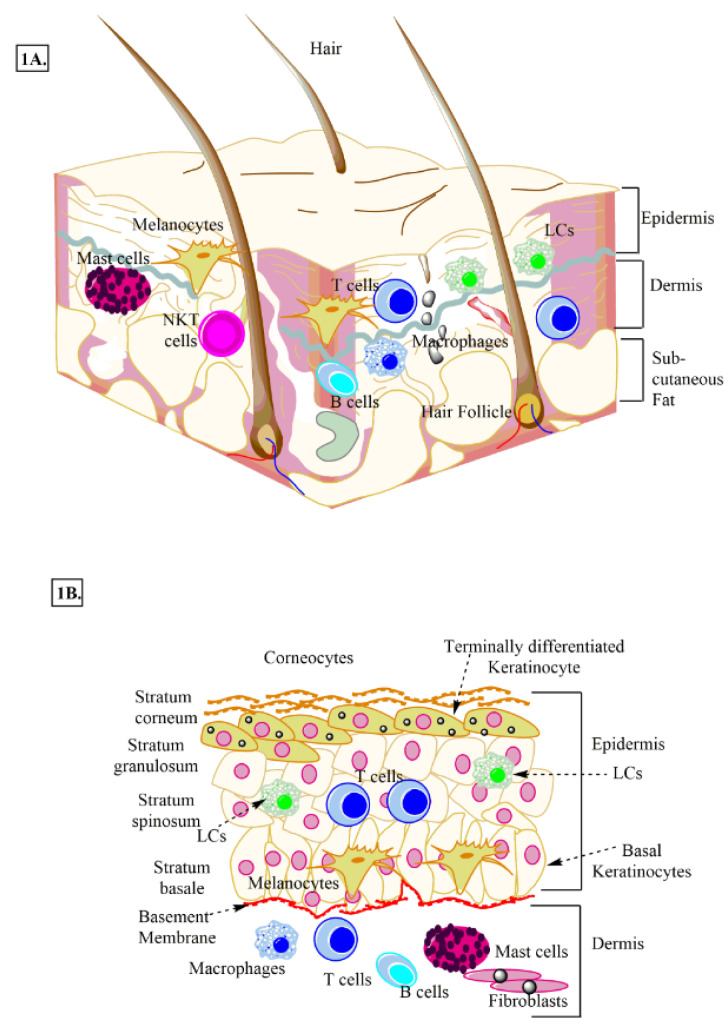
Schematic representation of human skin. A: Anatomical section of human skin showing epidermis, dermis, and adipose layer along with different immune cells and skin cells. B: Different layers of epidermis, stratum basale, stratum spinosum, stratum granulosum, and stratum corneum. LCs, melanocytes, keratinocytes and other immune cells (macrophages, T cells, NKT cells, and B cells etc.) present in the dermis and epidermis are also shown.

Skin is the largest immune organ that protects the host from outer dangers, including pathogens via serving as a barrier between internal organs and the outside environment (Salmon et al., 1994[177]). It comprises the skin-associated lymphoid tissue (SALT) (Lowes et al., 2014[121]). It harbors both innate (macrophages, DCs, ILCs, keratinocytes, Langerhans cells (LCs), melanocytes, γδT cells, and mast cells) and adaptive immune cells (T cells and B cells) that regulate its immune function (Figure 1A and 1B(Fig. 1)) (Egbuniwe et al., 2015[40]; Salmon et al., 1994[177]; Suwanpradid et al., 2017[199]). Skin immune cells (epithelial cells, keratinocytes, melanocytes, dendritic cells (DCs), mast cells, γδT cells, residential T and B cells, endothelial cells lining the skin microvasculature, and stromal cells, including fibroblasts and adipocytes) expressing PRRs, including TLRs play a crucial role in the host defense, immune homeostasis, and the generation of the pro-inflammatory immune response (Jameson and Havran, 2007[76]; Kumar, 2018[101]; Miller and Modlin, 2007[130]). Human skin has two components called epidermis and dermis (Figure 1A and 1B(Fig. 1)). The epidermis is the outer surface with four layers or strata. The stratum basale is the innermost (bottom) layer of the epidermis, and it comprises a single layer of undifferentiated epidermal cells called basal keratinocytes or keratinocyte stem cells, which divide very frequently and keep replenishing the stratum basale (Figure 1B(Fig. 1)) (Nestle et al., 2009[142]). 

Stratum spinosum (also called prickle cell layer) is present immediately above the stratum basale and differentiating basal keratinocytes to it to initiate their maturation process (Figure 1B(Fig. 1)). These keratinocytes change their shape from columnar to polygonal and produce keratin different from the one in stratum basale (Nestle et al., 2009[142]). Above stratum spinosum lies stratum granulosum, and dark clumps of cytoplasmic material called kerato-hyalin granules, which are highly basophilic (Figure 1B(Fig. 1)) characterize its keratinocytes.

These keratinocytes actively grow, make a stronger epidermal layer, and serve as the starting point of keratinization or cornification of the upper epidermal layer called stratum corneum. The stratum corneum is the uppermost or outermost layer of the epidermis that comprises degenerated keratinocytes, also called corneocytes, which are connected by desmosomes called corneosomes or corneodesmosomes (Figure 1B(Fig. 1)) (Jensen and Proksch, 2009[79]; Proksch et al., 2008[158]). During last stages, corneocytes lose their nucleus, get full with keratin, and protein and lipid layers surround them. The filaggrin and keratin comprise 80-90 % of the proteins of the stratum corneum (Proksch et al., 2008[158]). The filaggrin monomers aggregate keratin filaments to form tight keratin bundles (Nishifuji and Yoon, 2013[145]). Of note, stratum lucidum is present between stratum granulosum and stratum corneum in skin areas with high mechanical stress, including soles and palms. The skin epidermis, along with keratinocytes, contains melanocytes and LCs (Nestle et al., 2009[142]). CD8^+^T cells are present in stratum basale and stratum spinosum. Additionally, other immune cells, including macrophages, fibroblasts, nerve-related cells, DCs, mast cells, T cells, NK cells, and NKT cells, are also present in the epidermis (Figure 1A and 1B(Fig. 1)) (Nestle et al., 2009[142]). The lymphatic and vascular conduits drain the dermis of the skin to facilitate immune or inflammatory cell trafficking (Nguyen and Soulika, 2019[143]; Quaresma, 2019[159]).

The immunological roles of keratinocytes and γδT cells in skin immunity, including their role in orchestrating T cell immunity in the skin have been described somewhere else (Jiang et al., 2020[81]; Klicznik et al., 2018[96]; Macleod and Havran, 2011[124]). The prenatal skin expresses the same spectrum of TLRs as adult human skin (Iram et al., 2012[75]). TLRs 1-5 are highly expressed in the prenatal skin, and infants and children's skin express a higher level of TLR1 and TLR3 than the adult human skin (Iram et al., 2012[75]). However, neither DC precursors in the prenatal skin nor epidermal Langerhans cells and dermal DCs in the adult skin express TLR3 and TLR6. The staining pattern and intensity of both TLRs (TLR1 and TLR3) in fetal basal keratinocytes are similar to the keratinocytes of the adult skin (Iram et al., 2012[75]). 

The human *skin fibroblasts* express all TLRs from TLR1-TLR10 (Table 1(Tab. 1); References in Table 1: Akira et al., 2006[5]; Blasius and Beutler, 2010[9]; Das et al., 2016[33]; De Leo et al., 2016[35]; Fleer and Krediet, 2007[53]; Fore et al., 2020[54]; Henrick et al., 2019[67]; Nguyen et al., 2020[144]; Patidar et al., 2018[154]; Tartey and Takeuchi, 2017[204]; Tian et al., 2007[208]), and the activation of TLR1 and TLR2 produces higher levels of IL-6 and IL-8 than keratinocytes (Yao et al., 2015[226]). However, TLR10 in skin fibroblasts is non-functional. The UV-A exposure to the human fibroblasts increases TLR4 expression and associated downstream signaling molecules (MyD88 and NF-κB) that increase IL-6 and IL-8 cytokines along with matrix metalloproteinase 1 (MMP1) (Seo et al., 2018[185]). The TLR4 signaling inhibition inhibits the UV-A-mediated MMP1 and IL-8 secretion and ERK phosphorylation. Hence, TLR4 signaling on fibroblasts plays a crucial role in skin aging and senescence. *Human skin melanocytes* express fully functional TLRs, including TLR1, TLR2, TLR3, TLR4, TLR7, and TLR9 (Table 1(Tab. 1)), and their stimulation activates NF-κB-dependent pro-inflammatory immune response (IL-6, and IL-8, different chemokine production) (Yu et al., 2009[227]). The activation of these TLRs with lipopolysaccharide (LPS ligand for TLR4), PamCSK4 (TLR2 and TLR1), flagellin (TLR5 ligand), and Imiquimod (a TLR7 agonist) on melanocytes modulates pigmentation (Jin and Kang, 2010[83]). TLR9 activation in melanocytes controls melanogenesis (Sun et al., 2016[198]). Melanocytes in the skin also serve as immune cells and play a crucial role in skin immune response (Hong et al., 2015[70]). The human choroidal melanocytes also express functional TLRs (TLR1, TLR2, TLR3, TLR4, TLR5, and TLR6) and serve as innate immune cells (Cioanca et al., 2018[24], 2017[25]).

*The human LCs* in the epidermis maintain skin immune homeostasis through activating skin resident regulatory T cells (T_regs_) and mediate tolerance to self-antigens during steady-state, and during infection activate resident CD4+ memory T cells or effector memory T cells (located in the epidermis or at the papillary dermal-epidermal junction) producing IFN-γ and IL-17 also along with memory T_regs_ (Seneschal et al., 2012[184]). LCs also play a pro-inflammatory role in psoriasis, and their number increases in inflamed psoriatic skin (Borek et al., 2020[12]; Eaton et al., 2018[39]). The infiltrated LCs in psoriatic inflamed skin lesions highly express pro-inflammatory chemokines (CXCL1, CXCL10, CCL18, and CCL20) (Fujita et al., 2011[55]). The LCs isolated from human skin express TLR1, TLR2, TLR3, TLR5, TLR6, TLR7, and TLR10 (Table 1(Tab. 1)) (Flacher et al., 2006[52]; van der Aar et al., 2007[213]). However, human LCs do not respond to the LPS (a TLR4 ligand) and TLR7/8 ligands, indicating their absence or a low expression. On the other hand, murine LCs express TLR2, TLR4, and TLR9 but not TLR7, and their activation (except TLR4) produce IL-12p40 or Th1 cytokine polarizing cytokine and inhibits CCL17 or thymus and activation-regulated cytokine (TARC) (Mitsui et al., 2004[132]). Later studies have indicated the expression of TLR4 at the protein level in LCs and TLR5, TLR7, TLR8, and TLR9 at mRNA level (Wang X, 2011[218]). The exposure to UV rays increases the TRL2 and TLR4 expression in human LCs and upregulates downstream signaling molecules, including MAPK, NF-κB/p65, and IRF-3 (Wang et al., 2011[218]). Hence, UV rays modulate skin immunity via upregulating TLR2 and TLR4 expression in human LCs along with inducing vitamin D synthesis. However, the intradermal glucopyranosyl lipid A (GLA), a synthetic derivative of the lipid A tail of LPS oil in water emulsion (GLA-SE), is recognized by the TLR4 of the skin DCs, which promote the LCs emigration and local T cell activation (Schneider et al., 2012[182]).

The *human skin keratinocytes* express different TLRs (TLR1, TLR2, TLR3, TLR4 (only in immortalized human keratinocyte cell line HaCaT), TLR5, and TLR9 (Table 1(Tab. 1)) (Köllisch et al., 2005[97]; Lebre et al., 2007[108]). The stimulation of keratinocytes isolated from fetal, newborn, and adult skin with the TLR3 ligand called polyinosinic-cytidilic acid or poly I: C (analogous to viral double-stranded (ds) RNA) significantly enhances the CXCL8/IL-8, CXCL10/interferon-gamma induced protein 10 (IP-10), and TNF-α in the fetal and neonatal keratinocytes as compared to the adult keratinocytes. Hence, aging affects the TLR expression and the function in the human skin, indicating prenatal (*in utero* protection) and newborn skins have more potent TLR3-based antiviral immune response as compared to the adult humans compensating for the underdeveloped adaptive immune system. Keratinocytes also recognize dsRNA through retinoic acid-inducible gene-1 (RIG-1)-like receptors (RLRs), melanoma differentiation-associated protein 5 or MDA5/RIG-1, and protein kinase-R (PK-R) signaling to induce an antiviral immune response (Kalali et al., 2008[88]). The TLR3 activation in human keratinocytes increases the TLR7 expression as indicated by their response to the TLR7 agonist gardiquimod (a member of the imidazoquinoline antiviral immune response modifier family (Table 1(Tab. 1)) (Kalali et al., 2008[88]). Hence, the TLR3 activation in keratinocytes also regulates TRL7 expression. The stimulation of TLR3, 4, 5, and 9 in human keratinocytes stimulates the production of TNF-α, CXCL8, CCL2, and CCL20 (Lebre et al., 2007[108]). However, TLR3 (Poly I: C) and TLR9 (cytidine-phosphate-guanosine (CpG)-oligodeoxynucleotides or ODNs) stimulation also induce type 1 IFNs, CXCL9, and CXCL10 (Lebre et al., 2007[108]). The TLR5 (with flagellin) and TLR9 (polyI: C) activation selectively induce CCL27 production. The TLR3, TLR4, and TLR5 stimulation also upregulate intercellular adhesion molecule-1 (ICAM-1), HLA-DR, HLA-ABC, Fas receptor (FasR) or CD95, and CD40 expression (Lebre et al., 2007[108]). 

## TLRS in Different Skin-Inflammatory Conditions or Diseases

TLRs play a crucial role in the pathogenesis of different inflammatory and infectious diseases (Kumar, 2018[101]). They recognize different PAMPs or MAMPs and host-derived endogenous DAMPs such as heat shock proteins (Hsps), including Hsp60 and Hsp70, hyaluronic acid, and high mobility group box 1 (HMG-B1) protein. The recognition of various ligands by TLRs in microbial (bacterial, viral, fungal, and parasitic) infections or sterile tissue damage observed in acute trauma leads to the induction of inflammatory molecules including cytokines, chemokines, reactive oxygen species (ROS), and reactive nitrogen species (RNS) as well as the expression of chemokine receptors on innate immune cells (Fan and Malik, 2003[47]; Ghosh et al., 2006[59]; Sabroe et al., 2005[172]; Zeytun et al., 2010[229]). The prolonged activation of this inflammatory response may also predispose the host to chronic inflammatory conditions, including the development of cancer (Coussens and Werb, 2002[27]; Li et al., 2014[115]; O'Neill, 2008[148]). The activation of TLRs also plays a crucial role in neuroinflammation and neurodegenerative diseases that th author has discussed somewhere else (Kumar, 2019[103]). Psoriasin and CD24 play a crucial role in the differentiation of luminal mammary epithelial cells (MECs), and their dysregulation may play a crucial role in breast cancer development, including ductal carcinoma (Petersson et al., 2007[156]; Vegfors et al., 2012[216]). Thus, TLR signaling-induced production of S100A7 or psoriasin and S100A8/S100A9 (calprotectin) through TLR4-mediated NF-κB activation also plays a crucial role in breast cancer (Petersson et al., 2007[156]). Adalimumab, etanercept, and ustekinumab exert a protective effect in psoriasis via reducing the S100A7 and S100A8/S100A9 drastically in skin samples as indicated by immunohistochemistry (D'Amico et al., 2018[28], 2015[29]). TLR4 expression in the iris resident-macrophages and iris also plays a crucial role in acute anterior uveitis (AAU) during endotoxin (endotoxin-induced uveitis or EIU) challenge through MyD88-dependent NF-κB activation (Chen et al., 2009[23]; Li et al., 2010[113]). The TLR4, MyD88, and calprotectin signaling pathways also play a crucial role in the idiopathic acute anterior uveitis (IAAU) (Song et al., 2019[196]). Hence, understanding the role of TLRs in skin inflammation and inflammatory diseases becomes very crucial. The following section describes different skin-inflammatory conditions and the role of TLRs in their pathogenesis.

### Acne vulgaris 

TLR4 and TLR2 also play a crucial pro-inflammatory role in the pathogenesis of acne vulgaris (inflammation of hair follicles and accompanying sebaceous gland called inflammation of pilosebaceous units), which also involves *Propionibacterium acne* (*P. acne*) as a pathogen (Lai and Gallo, 2008[104]; McInturff and Kim, 2005[127]). *P. acne* is a Gram-positive bacterium, and TLR2 expressed on perifollicular and peribulbar macrophages recognizes them to initiate the pro-inflammatory immune response mediated by the NF-κB activation (IL-12 and IL-8), increases the TLR4 expression on keratinocytes, and increases the MMP9 secretion (Jugeau et al., 2005[85]; Kim et al., 2002[95]). The *Laurus nobilis* (*L. nobilis*) extract (LNE) exerts anti-inflammatory action via inhibiting the TLR signaling-induced NF-κB activation and the release of pro-inflammatory cytokines (IL-6, TNF-α) (Lee et al., 2019[109]). The bacteriocin-derived from *Enterococcus faecalis* (*E. faecalis*) called CBT-SL5 also inhibits IL-8 production from keratinocytes infected with *P. acne* due to the activation and increased expression of TLR2 and TLR4 (Lee et al., 2008[110]). The treatment with retinoic acid-derivatives, including isotretinoin that decreases the TLR2 expression during acne vulgaris and decreases skin inflammation (Kang et al., 2006[89]). Oral azithromycin (AZT) has also been shown effective in acne vulgaris (Kardeh et al., 2019[91]). AZT has a property to modulate TLR7 function. Hence, TLRs play a crucial role in the acne vulgaris pathogenesis, and their targeting may prove beneficial to the host.

### Atopic dermatitis (AD) or eczema

AD or eczema is a chronic relapsing skin-inflammatory condition derived in response to the defective terminal keratinocyte differentiation and a profound type 2 or Th2 immune response (Brunner et al., 2017[17]; Drucker et al., 2017[38]). It is different from psoriasis and also involves Th1, Th9, Th22, and Th17/IL-23 cytokine signaling events described in detail somewhere else (Brunner et al., 2017[17]; Esaki et al., 2016[44]; Yang et al., 2020[225]). It is highly prevalent in both children (15-25 %) and adults (4-7 %) in the USA and is a major health care burden (Esaki et al., 2016[44]; Silverberg, 2014[192], 2015[193]). TLRs are also crucial in AD pathogenesis. For example, TLR4 expression continuously increases from basal to upper spinous layers, including *stratum granulosum* (Panzer et al., 2014[150]). However, TLR2 expression decreases in macrophages in AD, increasing their tendency to get Gram-positive bacterial infection. A study in Italian children has indicated the prevalence of TLR2 R753Q (16 %) and TLR4-D299G (14.9 %) single nucleotide polymorphism (SNP) among AD patients (Salpietro et al., 2011[178]). However, TLR2 R753Q and A-16934T SNPs are not associated with higher susceptibility to AD in children in Turkey (Can et al., 2017[20]). Other genotyping studies in TLR2-16934A>T polymorphism performed in Japan indicates its association with AD and may serve as an indicator for its severity as TLR2-16934A>T polymorphism affects transcription activities associated with clinical findings (Potaczek et al., 2011[157]). In a Russia-based genome-wide association study (GWAS), TLR2 (p.Arg753Gln) and AD strongly associate with TLR4 (Asp299Gly) SNPs (Tyurin et al., 2017[211]). These patients exhibit dysregulated cytokine (IL-4 and IL-10) levels that well-correlate with imbalanced Th1, Th2, and Th17 immune responses. The meta-analysis study has further indicated the "GA" genotype of the TLR2 rs5743708 and "AG" genotype of the TLR4 rs4986790 may be associated with a high risk of AD in the Caucasian population (Zhang et al., 2019[230]). Hence, further studies in this direction are required to establish the population-specific impact of TLRs on AD. 

The stimulation of TLR2 in macrophages isolated from AD patients produce a low level of Th1/Th17 cytokines (IFN-γ, IL-12, and IL-17F) but a higher level of Th2 cytokine (IL-15) as compared to macrophages isolated from control human donors (Yang et al., 2020[225]). However, the continuous activation of TLR2 in AD patients promotes the pro-inflammatory Th1 immune response that increases inflammation at later stages. Certain *Candida spp. Malassezia* and other dermatophytes can infect and reside in atopic skin, and recognition of their PAMPs by TLRs (TLR1, TLR2, and TLR6) may further increase the inflammation (Faergemann, 2002[46]; Thammahong et al., 2020[206]; Yang et al., 2020[225]). Also, HSV-1 infection is common in AD patients, and TLR9 recognizing CpG-sequences of viral DNA may activate MyD88 and TRIF-dependent pro-inflammatory signaling that further enhances skin inflammation in AD patients (David and Longson, 1985[34]; Wollenberg et al., 2003[220]). Hence, TLRs play a crucial role in AD with or without infection. Further studies are required in the direction.

### Psoriasis

Psoriasis is a frequent inflammatory skin disorder categorized as an autoimmune disease. It is known as a disease of profound activation of T cells where abnormal activation of innate immune cells and pathogenic T cells cause skin inflammation and hyper-proliferation of keratinocytes causing erythematosus scaly plaques of variable sizes (Grän et al., 2020[61]). The pathogenic T cells in psoriasis comprise Th1, Th22, and Th17 cells, along with pathogenic pro-inflammatory cytokines IL-17, IL-22, IFN-γ, TNF-α, IL-12, IL-1, and IL-23 (Grän et al., 2020[61]; Lowes et al., 2014[121]). The details of psoriasis immunology have been discussed somewhere else (Albanesi, 2019[6]; Grän et al., 2020[61]; Lowes et al., 2014[121]). The UK-based GWAS study has indicated the strong association between a missense variant rs4986790 of *TLR4* (Asp229Gly) and plaque-type psoriasis and the early onset of the disease (Smith et al., 2016[194]). Also, a Poland-based GWAS has shown a strong association between a high (> 10) Psoriasis Area Severity Index (PASI) and people with TT -1237 T/C TLR9 genotype polymorphism (Zabłotna et al., 2017[228]). The TT genotype of TLR9 polymorphism also has the PASI score >15 more frequently than the control population. Further studies in different populations are needed in the direction for concluding as this study indicates that TLR2 and TLR9 polymorphism may impact only the clinical symptoms of psoriasis but not their pathogenic role in psoriasis development (Zabłotna et al., 2017[228]). However, a study has indicated a significant relationship between TLR9-1486T/C SNP variants and responsiveness to the standard narrow-band ultraviolet B (NBUVB) therapy (Romaní et al., 2015[167]). According to this study, the patients with TC and CC genotype show a better improvement in the PASI score and skin inflammation as compared to patients with TT genotype. A study conducted in the Chinese population has indicated the TLR2 SNP (rs3804099) association with the heritability of psoriasis vulgaris (Shi et al., 2016[191]). However, in the Turkish population, the TLR2 SNP (rs4696480) is strongly associated with the heritability of psoriasis (Sabah-Özcan and Gürel, 2019[171]). Hence, population genetics impacts TLRs and their impact on the incidence, pathogenesis, and clinical outcome of the disease. However, further studies are required to establish these findings in other populations and different TLRs.

The exposure of the TLR7 and TLR8 agonist Aldara (5 % imiquimod) to the skin induces psoriasis plaque spreading via inducing IL-23, IL-17A, and IL-17F in the epidermis (Horváth et al., 2019[71]; van der Fits et al., 2009[214]). Imiquimod has also been used to develop a mouse model of psoriatic itch (Sakai et al., 2016[174]). Mice lacking IL-17 or IL-23 receptors do not produce psoriasis plaques upon exposure to the Aldara, indicating the importance of the IL-17/IL-23 axis. Further studies have shown that the deletion of IL-17R on T cells and the myeloid cell does not impact the disease development (Moos et al., 2019[136]). However, the IL-17RA (IL-17 receptor A or CDw217) deletion in keratinocytes fully protects the animals from psoriasis development upon topical application of the Aldara. These mice have infiltrated monocytes in the skin but lacked pro-inflammatory neutrophils (Moos et al., 2019[136]). IL-17A recognition by IL-17R expressed on keratinocytes plays a crucial role in the psoriasis development and neutrophil infiltration in experimental models. Also, the TLR7/ TLR8 and TLR9 activation during psoriasis promotes the M1 macrophage or inflammatory macrophage (IM) polarization, which further amplifies the skin inflammation (Lu et al., 2018[122]). 

Aldara that is prescribed to humans for actinic keratosis or genital and perianal warts caused by human papillomavirus (HPV) may induce psoriasis (Szeimies et al., 2004[201]; van der Fits et al., 2009[214]). A 74 years old man receiving a treatment with 5 % imiquimod for two weeks to treat actinic keratitis had developed psoriasis (Machler et al., 2011[123]). The treatment with AZT in mice with 5 % imiquimod or Aldara-induced psoriasis improves skin inflammation. This effect involves DC function modulation through a decrease in the lysosomal acidification required for TLR7 maturation and signaling (Huang et al., 2016[73]). The AZT treatment reduces the level of several pro-inflammatory cytokines (IL-17, IL-22, and IL-23) and accumulation of DCs, CD4^+^T cells, CD8^+^T cells, and Th17 cells in the skin of mice suffering from imiquimod-induced psoriasis. Hence, AZT may have the potential for psoriasis therapeutics through modulating TLR7 expression and function in skin DCs. Further studies should be performed in other skin resident cells (LCs, melanocytes, and keratinocytes) expressing TLR7 upon TLR3 activation during psoriasis with AZT.

The TLR2 and TLR4 DAMP called Hsp60 level increases in the skin of both guttate (a type of psoriasis seen in children and young adults, which shows small, red, scaly, and teardrop spots on the skin but do not leave a scar) and plaque psoriasis (Seung et al., 2007[188]). However, TLR4 expression increases in both forms of psoriasis. The skin from guttate psoriasis patients has a higher TLR4 expression than plaque psoriasis patients and healthy donors (HDs) (Seung et al., 2007[188]). The increased LL-37 or cathelicidin (an antimicrobial peptide or AMP) expression in psoriatic skin serves as an auto-antigen to activated T cells, which produce pro-inflammatory IFN-γ and IL-17 (Lande et al., 2014[105]). However, along with acting as an auto-antigen, LL-37 also binds to the inert self-DNA that is recognized by the endosomal TLR9 of plasmacytoid DCs (pDCs) to produce type 1 IFNs (Lande et al., 2007[106]). LL-37 also binds to self-RNA in psoriasis patients, and TLR7/TLR8 of classical myeloid DCs recognize this complex (Ganguly et al., 2009[57]). This recognition produces IL-6 and TNF-α and induces the maturation of DCs. Hence, TLRs of DCs play a crucial role in the aggravation of skin inflammation in psoriasis patients. However, it will be interesting to observe the recognition of the LL-37-self-DNA and LL-37-self-RNA complex by the TLRs expressed on keratinocytes and LCs. Also, keratinocytes express LL-37 (Tonel and Conrad, 2009[209]).

Keratinocytes present in the skin expressing TLR5 recognizes flagellin of bacteria, including *Escherichia coli* (*E. coli*) enhances the S100A7 (psoriasin) and S100A8 (calgranulin A)/S100A9 (calgranulin B), an antimicrobial heterodimeric complex also called calprotectin expression (Abtin et al., 2010[1], 2008[2]). The overexpression of S100A7 (psoriasin) in keratinocytes dysregulates the epidermal cell or epidermis differentiation in psoriasis patients (Ekman et al., 2017[43]). The involucrin, desmoglein 1, transglutaminase 1, and CD24 expression dysregulation indicates this (Ekman et al., 2017[43]). Psoriasis patients also show increased circulating levels of HMGB1 and psoriasin (Borsky et al., 2020[13]). The human psoriasin induces the IL-1α production in a RAGE (receptor for advanced glycation end products)-p38 MAPK and calpain-1-dependent manner that increases the thickness of psoriatic skin (Lei et al., 2017[111]). Also, calprotectin (S100A8/ S100A9) upregulated in the inflamed skin of psoriatic patients serves as endogenous ligand for TLR4, and their binding further enhances inflammation in psoriasis patients (D'Amico et al., 2018[28]; Ehrchen et al., 2009[41]). Normal human skin either does not have S100A8/S100A9 or has a negligible level. A missense variant rs4986790 of TLR4 (Asp229Gly) has shown a strong association with the early onset of psoriasis and the plaque type (Smith et al., 2016[194]). A penetratin-conjugated small peptide (TIP3) derived from the core of β-sheet region of Toll/interleukin-1 receptor domain-containing adapter protein (TIRAP) blocks both MyD88 (myeloid-differentiation 88) and TIR-domain containing Adaptor inducing interferon-β (TRIF)-dependent TLR4 and TLR3 downstream signaling pathways and ameliorates the inflammatory symptoms in experimental psoriasis animal models (Achek et al., 2020[3]). A decoy peptide called MIP2 designed from the αC helix of TIRAP also blocks both MyD88 and TRIF-dependent TLR4 downstream signaling to exert a protective action in the psoriasis animal model (Shah et al., 2020[189]).

Also, microRNA-181b (miR-181b) levels are low in the human epidermal keratinocytes (HEKs) of psoriatic skin lesions as compared to the skin of normal humans (Feng et al., 2017[48]). The miR-181b targets TLR4 indicating that due to its low level, TLR4 expression increases in the inflamed skin of the psoriasis patients that further aggravates the inflammation via recognizing different DAMPs (Feng et al., 2017[48]). Also, miR-181b-5p inhibits keratinocyte proliferation in psoriasis via targeting Akt3 (AKT serine/threonine kinase 3) (Zheng et al., 2019[231]). However, a study has indicated that Akt regulates TLR3 and TLR4-mediated signaling via regulating TRIF-dependent signaling through interacting with the TANK-binding kinase 1 (TBKI) (Joung et al., 2011[84]). Hence, Akt activation via TBK1 regulates TLR3 and TLR4-mediated type 1 IFN release. Thus miR-181b plays a crucial role in psoriasis via directly regulating the TLR4 expression and its function through Akt. We require further studies in this direction.

TLR5 and TLR9 expression also increase in hyper-proliferative psoriasis skin keratinocytes in the presence of TGF-α (Miller et al., 2005[131]). The LCs infiltrated in the inflamed psoriasis skin also produce the pro-inflammatory IL-23 cytokine in response to the TLR activation (TLR2 (Zymosan, a fungal PAMP), TLR4 and TLR7/TLR8) that further increases in the presence of TNF-α (Martini et al., 2017[126]; Sweeney et al., 2016[200]). IL-23 is considered a potential drug target for autoinflammatory immune diseases, including psoriasis (Tang et al., 2012[203]). Hence, they may contribute to the local inflammation by recognizing the local DAMPs and PAMPs to aggravate the psoriatic skin inflammation through different TLRs expressed. This pro-inflammatory action of LCs may further promote Th17 cells development and activation to aggravate skin inflammation psoriasis (Sweeney et al., 2016[200]). Human β-defensin 3 (HBD3) enhances IL-23 production from LCs in response to TLR stimulation (Sweeney et al., 2016[200]). HBD3 levels are increased in psoriasis patients. Hence, LC TLR signaling also plays a crucial role in psoriasis. The details of LCs in psoriasis is beyond the topic of the current review and has been discussed somewhere else (Eidsmo and Martini, 2018[42]). Hence, TLRs expressed on different skin cells, keratinocytes, LCs, and DCs play a crucial role in psoriasis and associated inflammatory phenotype.

### Skin cancer

Skin cancer may be of different types, including melanoma (comprising melanocytes and melanin) and non-melanoma skin cancer (NMSC), which include basal cell carcinoma (BCC) and squamous cell carcinoma (SCC). NMSCs or keratinocyte carcinomas are the most common skin cancer in the USA, which are continuously increasing in number (Rogers et al., 2015[165], 2010[166]). However, both BCCs and SCCs equally account for the Medicare population in the USA (Rogers et al., 2015[165]). Cutaneous melanoma is the most common melanoma and accounts for more than 90 % of all melanoma cases (Chang et al., 1998[21]). It is the 19^th^ most cancer around the world, and the incidence of melanoma is increasing continuously worldwide (Bray et al., 2018[14]; Ferlay et al., 2010[49]). Both keratinocytes and melanocytes are the crucial skin cells giving rise to skin cancers, and both express different TLRs described earlier, and these TLRs (TLR2, -3, -4, -7, and -9) further increase during skin cancer, including melanoma (Coati et al., 2016[26]; Goto et al., 2008[60]; Mittal et al., 2010[133]; Saint-Jean et al., 2011[173]). The TLR stimulation by different PAMPs and DAMPs (HMGB1 released from necrotized keratinocytes) induces NF-κB-dependent pro-inflammatory immune response (IL-6, IL-18, TNF-α, and immunosuppressive IL-10), inflammatory infiltrate infiltration, and the chronic inflammation may predispose the host to cancer (Mittal et al., 2010[133]; Sato et al., 2009[180]). The TLR9 activation on melanocytes regulates melanogenesis via NF-κB activation and thus may have a role in hypo or hyperpigmentation (Sun et al., 2016[198]). Hence, it will be interesting to investigate the TLR9 activation in melanocytes in patients with melanoma.

The keratinocytes in NMSCs or melanocytes in melanoma expressing TLRs can evade the immunosurveillance and promote the tumor or cancer development (Burns and Yusuf, 2014[19]). The TLR4 signaling inhibition through icariside II (a herbal product isolated from *Herba Epimedii*) in human melanoma A375 cells decreases pro-inflammatory cytokine levels along with the vascular endothelial growth factor (VEGF), an essential element for tumor-associated angiogenesis and induces their apoptosis (Wu et al., 2012[221], 2013[222]). Also, the treatment with pioglitazone, a peroxisome proliferator-activated receptor (PPAR)-γ agonist inhibits the TLR4-mediated pro-inflammatory signaling, MyD88-mediated NF-κB activation in melanoma cells *in vitro* and suppresses melanoma *in vivo* (Dana et al., 2019[32], 2020[31]). Fenofibrate, a PPARα ligand, exerts an anti-melanoma effect via inhibiting TLR4 signaling in melanoma both *in vitro* and *in vivo* (Dana et al., 2020[30]). Other TLR4 antagonists, including eritoran, (+)-naloxone, ST2825, and resatorvid, should have also shown beneficial effects in skin cancers, including melanoma (Blohm-Mangone et al., 2018[10]; Dickinson and Wondrak, 2018[37]; Janda et al., 2016[77]). 

The poly I: C-induced TLR3 activation on immune cells exerts antitumor action through increasing antitumor effects of T cell immunity as indicated by the increased production of IFN-γ and TNF-α (Salem et al., 2005[176]). Different TLR3 agonists, including Hiltonol (polyriboinosinic-polyribocytidylic acidpolylysine carboxymethylcellulose or poly I: CLC), are under clinical trial for melanoma and other cancers (Le Naour et al., 2020[107]). Hence, activating TLR3 during skin cancer may have a protective effect. The TLR7/TLR8 activator Aldara or 5 % imiquimod, has been approved for skin cancers, including BCCs, and has been found beneficial in metastatic melanoma (Coati et al., 2016[26]; Navi and Huntley, 2004[141]). Also, resiquimod is another TLR7//TLR8 agonist that is ten times more potent to induce Th1 immune response than imiquimod and can be considered as a potent vaccine adjuvant (due to a high increase in the antigenicity of the cancer antigen) against cancers, including melanoma with the vaccine candidate NY-ESO-1 protein, emulsified Montanide (Sabado et al., 2015[170]; Thomsen et al., 2004[207]). Even its topical application helps in the regression of cutaneous T-cell lymphoma or CTCL (malignant T cells accumulate in the chronically inflamed skin) due to the TLR7-mediated activation of DCs, increased skin T cell effector functions, and the increased cytolytic action of the natural killer or NK cells (Brunner et al., 2020[18]; Rook et al., 2015[168]; Stolearenco et al., 2020[197]). The TLR2, TLR4, and TLR9 expression on keratinocytes express in inflamed skin of patients with CTCL, including both Mycosis fungoides (MF) and Sezary syndrome (SS, an aggressive form of CTCL) (Jarrousse et al., 2006[78]). Additionally, TLR9 activation via their agonists (CpG ODN or CpG B-type oligodeoxynucleotide) and other compounds, including PF-3512767 through intralesional subcutaneous injection, have shown a protective effect in melanoma and BCCs through activating skin innate immune cells, including DCs and LCs, which activate melanoma-specific CD8^+^T cells and NK cells, which kill cancerous cells through their cytotoxic action (Hofmann et al., 2008[68]; Krieg, 2008[98]; Molenkamp et al., 2008[134], 2007[135]; Pashenkov et al., 2006[153]). The treatment with PF-351276 increases the IL-6, IL-12p40 (activates NK cells and their cytotoxic function), IP-10, and TNF-α (Hofmann et al., 2008[68]; Pashenkov et al., 2006[153]). TLR9 activation decreases the population of immunosuppressive T_regs _(Molenkamp et al., 2007[135]). Thus TLR3, TLR7/TLR8, and TLR9 activation are crucial therapeutic and adjuvant-based vaccine-oriented approaches to target skin cancers. Hence, both the activation and inhibition of TLRs (depending on their type and role) may prove helpful in targeting skin inflammation and cancer. 

## TLR Signaling Involved in the Generation of Inflammatory Immune Response during Skin Inflammation and Inflammatory Diseases

**Figure 2:**
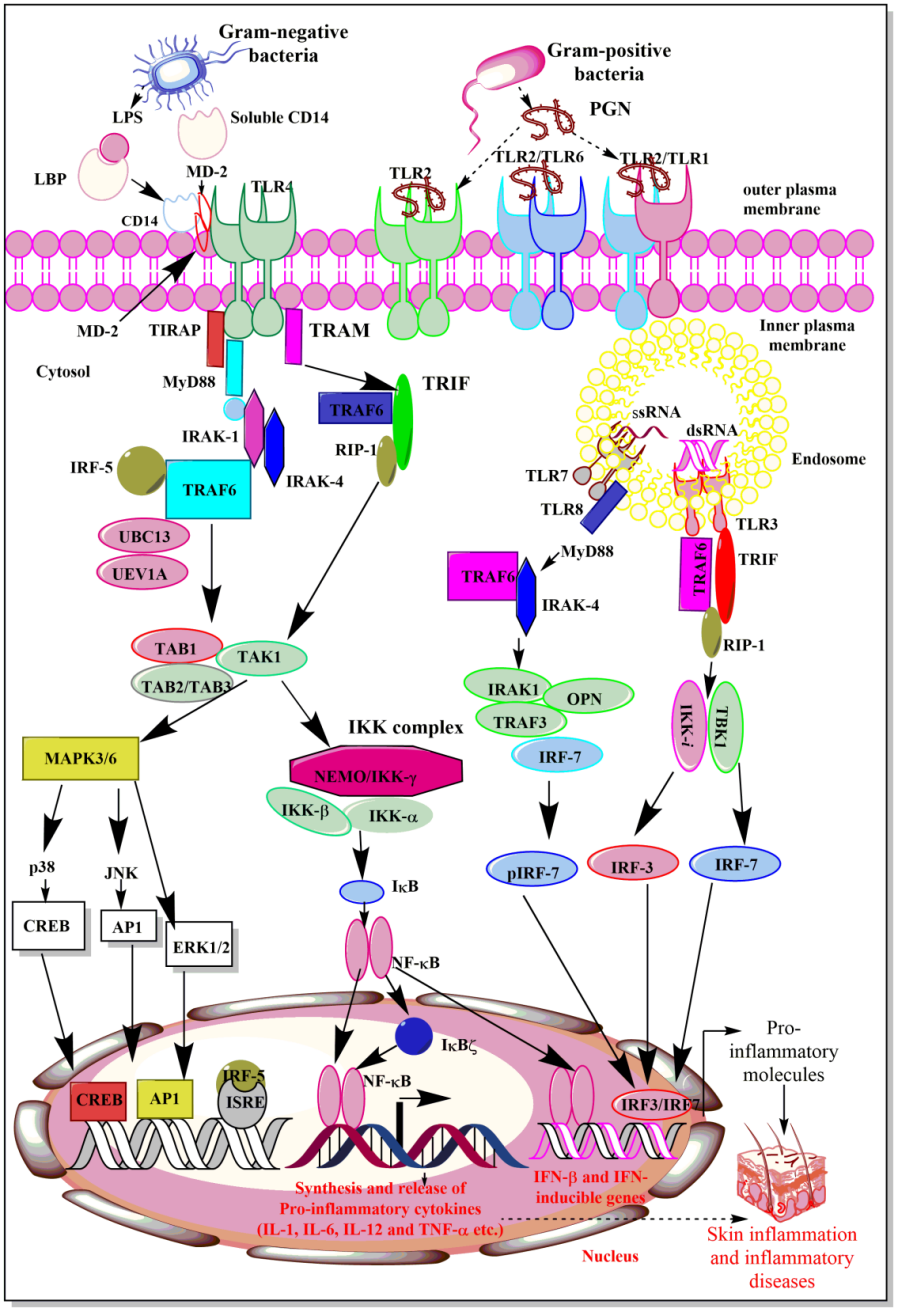
Schematic representation of TLR4, TLR7/TLR8, and TLR9-dependent signaling pathways. TLR 4 activation induces NF-κB activation through both MyD88-dependent and MyD88-independent but TRIF-dependent signaling pathways. Whereas, TLR7/TLR8 activation upon binding to their corresponding ligands induces MyD88-dependent signaling pathways to induce IRF7-dependent IFN synthesis, which have potent immunomodulatory action on both innate and adaptive immune cells. The TLR9 activation upon recognizing and binding to its ligand involves TRIF-dependent signaling pathway for IFN synthesis. The details are mentioned in the text.

The recognition of bacterial PAMPs such as LPS, lipoteichoic acid (LTA), peptidoglycan (PGN), porins, flagellin, and CpG-DNA by their corresponding TLRs (TLR4, TLR2, TLR5, TLR9) induces the events, which converge at the activation of NF-κB, which activates genes responsible for the synthesis and release of pro-inflammatory mediators including cytokines, chemokines, ROS and RNS (Figure 2(Fig. 2)) (Akira and Takeda, 2004[4]; Akira et al., 2006[5]; Feuillet et al., 2006[50]). TLR5 physically interacts with TLR4 that biases the signaling pathway towards MyD88-dependent by forming Myddosome (a complex of MyD88 and IRAK4) to activate NF-κB and associated downstream pro-inflammatory genes, including cytokines (Hussain et al., 2020[74]). The deficiency of TLR5 alters the response to the TLR4 stimulation. For example, people with a dominant-negative *TLR5* single nucleotide polymorphism (SNP) (rs5744168) for the common stop codon polymorphism in the ligand-binding domain of the TLR5 (*TLR5392STOP*) do not respond to the flagellin and are more prone to *Legionella pneumophila pneumonia* (Hawn et al., 2003[65]). The impact of dominant-negative *TLR5* SNP (rs5744168) needs further studies on these people. Macrophages isolated from rs5744168 minor-allele carriers also show a defective response to TLR4 activation, but not to TLR2, TLR3, and TLR9 (Hussain et al., 2020[74]).

The recognition of LPS by TLR4 involves the binding of circulating LPS to LBP (LPS-binding protein) and transfers LPS to CD14, a glycophosphatidylinositol (GPI)-anchored membrane protein (Figure 2(Fig. 2)). Of note, CD14 also exists in its soluble form that is also capable of binding the LPS-LBP complex and is responsible for the recognition of smooth LPS, but not for rough LPS (Jiang et al., 2005[82]). Membrane-bound CD14 lacks an intracellular domain and does not exhibit a downstream signaling event. It complexes with TLR4 to form a functional LPS receptor complex. This binding of LPS to TLR4 also requires an MD-2 molecule, which associates itself with the extracellular domain of TLR4 (Park et al., 2009[151]). Thus, the active LPS receptor complex comprises TLR4, CD14, and MD-2, where CD14 and LBP are responsible for enhancing TLR4-dependent LPS signaling and cell stimulation (Lizundia et al., 2008[119]). Once the extracellular binding of LPS to TLR4 is complete, it leads to the downstream signaling involving various adaptor molecules, kinases, phosphatases, and activation of the NF-κB transcription factors. 

The NF-κB activation induces the expression and release of several pro-inflammatory cytokines (TNF-α, IL-1α, IL-6, and IL-8) and other molecules responsible for the generation of so-called cytokine storm, systemic inflammation, and development of sepsis (Figure 2(Fig. 2)). This rapid LPS detection by TLR4 forms a Supramolecular Organizing Center (SMOC) called myddosomes (Bonham et al., 2014[11]; Kagan et al., 2014[87]; Lin et al., 2010[116]; Motshwene et al., 2009[138]). This myddosome comprises MyD88, TIRAP (an adaptor molecule), and several serine-threonine kinases of the interleukin-1 receptor-associated kinase (IRAK) family (Rosadini and Kagan, 2017[169]). Myddoomes act as the sub-membranous organizing center and directs TLR4-initiated signaling to promote NF-κB and AP-1 (activator/activating protein-1) activation leading to the expression of pro-inflammatory genes (Rosadini and Kagan, 2017[169]). TLR downstream signaling pathways have been well described elsewhere (Akira and Takeda, 2004[4]; Kawai and Akira, 2010[92]; Tartey and Takeuchi, 2017[204]). The following section describes the overview of TLR signaling pathways.

### MyD88-dependent and -independent TLR4 mediated intracellular signaling 

The formation of LPS, CD14, MD-2, and TLR4 complex induces conformational changes in TLR4 molecule due to its homodimerization via interaction between their intracellular TIR-domains (Vaure and Liu, 2014[215]). The change causes recruitment of TIR-domain containing adaptor molecules towards the cytoplasmic domain of the TLR4 homodimer through homophilic interactions between TIR-domains (Vaure and Liu, 2014[215]). At least 5 TIR-domain-containing adaptor molecules from two different pathways play a direct role in TLR4 signaling. They include 1) MyD88. 2) MyD88-Adaptor-like (MAL) protein, also called TIR-domain containing Adaptor protein (TIRAP), 3) TIR-domain containing Adaptor inducing interferon-β (TRIF) or TIR-domain containing Adaptor molecule-1 (TICAM-1), 4) TRIF-related Adaptor molecule (TRAM) or TIR-domain containing protein (TIRP) or TIR-containing Adaptor molecule-2 (TICAM-2) and 5) sterile α-and armadillo-motif-containing protein (SARM) (O'Neill and Bowie, 2007[147]; Vaure and Liu, 2014[215]). TLR4 requires these adaptor proteins to mount a comprehensive immune response against a pathogen. The adaptor proteins, TIRAP and TRAM, are also called sorting adaptors. They interact with phosphoinositides (PIs) for promoting LPS induced pro-inflammatory signaling and define the locale for the formation of signaling complexes at specific subcellular sites (Kagan, 2012[86]). These *sorting adaptors* are specific regulatory factors for TLR4 signaling in the sense that they are present at the site of their requirement (for example, TIRAP is located in phosphatidylinositol 4,5 bisphosphate (PIP2)-rich regions of the cell surface (inner leaflet of plasma membrane) and TRAM is located on plasma membrane and endosomes). They recruit the downstream signaling mediators to their sites and define the type of signaling pathway initiated from that location (Rosadini and Kagan, 2017[169]). They do so through binding to PIs found on cellular organelles such as the inner surface of the cellular plasma membrane or endosomes, the eventual sites of TLR4 signal transduction (Rosadini and Kagan, 2017[169]). These sorting adaptors recognize the dimerized TLR4 at the cell surface or on endosomes to form myddosomes or TRIF signaling complexes and promote inflammatory signaling (Rosadini and Kagan, 2017[169]). Thus PI(4,5)P2 plays a crucial role in initiating TLR4 signaling at the plasma membrane, and its modulation has the potential to modulate the TLR4 pathway (Rosadini and Kagan, 2017[169]). 

The intracellular signaling pathway of TLR4 signaling activates two main pathways:

### MyD88-TIRAP dependent signaling pathway: 

It is mainly involved in the early regulation of NF-κB activation and production of pro-inflammatory cytokines (IL-6, IL-12, and TNF-α) (Figure 2(Fig. 2)). MyD88 was first named in the 1990s as a protein that was induced during the terminal differentiation of M1D^+^ myeloid precursors in response to IL-6; the 'MyD' stands for myeloid differentiation and '88' depicts the gene number in the list of induced genes (Lord et al., 1990[120]). The MyD88-dependent pathway mainly involves its recruitment to the cytoplasmic portion of TLR4 via TIR domains, and in turn, recruits IL-1R-associated kinase-4 (IRAK-4) and IRAK-1 via death domains (Figure 2(Fig. 2)). Chlorogenic acid (CGA), an important anti-inflammatory constituent of *Lonicerae flos* extract inhibits IRAK-4 to prevent pro-inflammatory cytokines (IL-6, IL-8, IL-1, TNF-α, and HMG-B1) generation in response to the TLR signaling-mediated activation of NF-κB/AP-1 activation (Park et al., 2015[152]). After recruitment of IRAK-1 to MyD88, the former phosphorylates via activated IRAK-4 and complexes with TRAF-6 (TNFR- associated factor 6), a RING (really interesting new gene) finger-domain E3 ubiquitin ligase (Li et al., 2002[114]). Further, TRAF-6, in association with ubiquitination E2 enzyme complex, which comprises UBC13 (ubiquitin-conjugating enzyme E2 13) and UEV1A (ubiquitin-conjugating enzyme E2 variant 1A), catalyzes the K63-linked polyubiquitin chain formation on TRAF6 itself and IKK-γ/NF-κB essential modulatory (NEMO) (Figure 2(Fig. 2)) (Deng et al., 2000[36]). 

TRAF-6 also recruits a complex comprising a MAPK kinase kinase (MAP3K) named TGF-β-activated kinase 1 (TAK1) and the TAK1 binding proteins, TAB1, TAB2, and TAB3 (Figure 2(Fig. 2)) (Arthur and Ley, 2013[8]; Wang et al., 2001[217]). TAK1 then phosphorylates IKK-β and MAP kinase kinase 6 (MKK6), and other MAP-2K, and modulates the activation of NF-κB and MAP kinases, inducing genes responsible for inflammation and associated tissue/organ damage. Additionally, TAK1 or MAP3K7 can directly stimulate MAPK kinase (MAP2K) for p38 and JNK (c-Jun N-terminal kinase) and acts as a crucial MAP3K for ERK1/2, p38, and JNK activation downstream to TLR signaling causing the activation of the activator protein-1 (AP-1) family of transcription factors (TFs) or stabilization of mRNA regulating pro-inflammatory immune response (Figure 2(Fig. 2)) (Akira et al., 2006[5]; Kawai and Akira, 2010[92]; Sakurai, 2012[175]; Wang et al., 2001[217]). Thus, TAK1 plays an important role in TLR signaling, along with TNF and IL-1 signaling pathways (Sato et al., 2005[179]) (Figure 2(Fig. 2)). However, along with NF-κB gene activation, the transcription factor IRF5 also regulates the expression and release of IL-6, IL-12, and TNF-α (Takaoka et al., 2005[202]). Upon stimulation of innate immune cells with TLR4 ligands, interferon regulatory factor-5 (IRF5) translocates to the nucleus and binds to potential IFN-stimulated response element (ISRE) motifs present in the promoter regions of cytokine genes. IRF5 activation is also associated with pro-inflammatory M1 macrophage polarization and thus induces pro-inflammatory cytokine release, and its inhibition prevents inflammatory damage during sepsis and other inflammatory diseases (Wei et al., 2019[219]). Due to the less IRF5 expression in newborn macrophages upon LPS-mediated TLR4 6-7 fold, lower levels of TNF-α production occurs as compared to the adult macrophages (Schneider et al., 2018[181]).

Additionally, IκBζ [also known as Molecule possessing ankyrin repeats induced by lipopolysaccharide (MAIL) and IL-1 inducible nuclear ankyrin-repeat protein (INAP)] is an ankyrin-repeat-containing nuclear protein that is highly homologous to the IκB family member B cell lymphoma-3 (Bcl-3) and is hard to detect in resting cells. However, it upregulates upon TLR4 stimulation and with IL-1β treatment (Muta et al., 2003[140]; Yamamoto et al., 2004[223]). IκBζ stimulates the synthesis and release of IL-6, IL-12, and other pro-inflammatory genes (Yamamoto et al., 2004[223]). Unlike IκB-α and IκB-β, IκBζ is located in the nucleus and binds to the p50 subunit of NF-κB and is recruited to the NF-κB binding site of the IL-6 promoter and regulates the transcriptional activity of NF-κB in both pro-inflammatory and anti-inflammatory manners depending on activation or suppression of the genes involved in the process of inflammation (Motoyama et al., 2005[137]; Muta, 2006[139]; Muta et al., 2003[140]; Yamamoto et al., 2004[223]) (Figure 2(Fig. 2)). Thus, this IκBζ activation occurs downstream of MyD88 and TRAF6 signaling pathways upon activation of TLR4 (and other TLR)-induced signaling (Eto et al., 2003[45]). The IκBζ activation occurs rapidly upon TLR4 stimulation by its ligands and regulates the expression of genes involved in the functioning of the innate immune response in microbial infections.

### MyD88-independent or TRIF-TRAM-dependent signaling pathway: 

The TRIF-TRAM-dependent pathway gets activated in macrophages and dendritic cells (DCs) upon ligand-specific stimulation of TLR4 (Figure 2(Fig. 2)). In addition to TLR4, this pathway also gets activated following stimulation of TLR3, TLR7, and TLR9 upon their ligand-specific stimulation (Figure 2(Fig. 2)). However, TLR2 stimulation does not initiate this pathway. TRIF-TRAM pathway activates IRF3, NF-κB, and MAP kinases, which upregulate genes for type 1 interferons (IFNs), co-stimulatory molecules, and pro-inflammatory cytokines (TNF-α, IL-6, IL-8, and IL-12) (Figure 2(Fig. 2)). This TLR4 and LPS mediated activation of the MyD88-independent pathway involves the association of TRAM with TLR4 and TRIM by acting as a bridge between TLR4 and TRIF (Akira et al., 2006[5]). Further, TRIF interacts with TRAF6 and the receptor-interacting protein (RIP)-1, which is involved in TLR3-mediated stimulation of NF-κB (Figure 2(Fig. 2)) (Meylan et al., 2004[129]). This interaction of TRAF6 with the N-terminal TRAF-binding domain of TRIF activates TAK1 through similar mechanisms involved in the MyD88-dependent signaling pathway. However, the TRIF interaction with receptor-interacting serine/threonine-protein kinase 1 (RIP1) causes K63-linked polyubiquitination of the TAK1 (Figure 2(Fig. 2)). Furthermore, RIP1 also interacts with TNF receptor type 1-associated death domain protein (TRADD), and this multiprotein complex is actively involved in NF-κB activation. 

TRIF also activates TRAF-family-member-associated NF-κB activator (TANK) binding kinase 1 [TBK1 or TRAF2-associated kinase (T2K) or NF-κB activating kinase (NAK)] through TRAF3, which is crucial for inducing type 1 interferons (IFNs) and anti-inflammatory cytokine IL-10 (Figure 2(Fig. 2)) (Hacker et al., 2006[64]; Oganesyan et al., 2006[146]). Activated TBK1/IKK-**i **(also named as IKK-ε)] complex phosphorylates IRF3 and IRF7 (Figure 2(Fig. 2)) (Fitzgerald et al., 2003[51]; Sharma et al., 2003[190]). TBKI, along with IKK-**i **plays a crucial role in TRIF-mediated IFN response (Hemmi et al., 2004[66]; McWhirter et al., 2004[128]; Perry et al., 2004[155]). These kinases phosphorylate monomeric IRF3 and IRF7, which form homodimers, translocate into the nucleus, bind to ISREs, and induce IFN gene expression (Akira et al., 2006[5]). Additionally, the Pellino family of E3 ubiquitin ligases also plays a crucial role in effective TLR signaling (Jiang and Chen, 2011[80]). Pellino-1 deficiency causes a defective TRIF-dependent NF-κB activation and pro-inflammatory cytokine production (Chang et al., 2009[22]). Now it is well documented that TBK1/IKK-**i** also phosphorylate Pellino-1, which in turn ubiquitinates RIP-1 and impacts TRIF-dependent NF-κB activation by recruiting RIP-1 (Kawasaki and Kawai, 2014[94]). Furthermore, Pellino-1 also regulates IRF3 activity through DEAF-1, a transcription factor that facilitates the binding of IRF3 to the IFN-β promoter (Jiang and Chen, 2011[80]). 

In addition to Pellino-1, inositol lipid called phosphatidylinositol 5 phosphate (PtdIns5P) also regulates IRF3 activation (Kawasaki and Kawai, 2014[94]). PtdIns5P ligates with both IRF3 and TBK1 to facilitate the TBK1 and IRF3 complex formation, which phosphorylates IRF3 (Kawasaki and Kawai, 2014[94]). Thus, these signals are required not only for the effective innate immune response to clear the pathogen via inducing the expression of genes required for a pro-inflammatory immune response but also provide future instruction for adaptive immune response through the release of cytokines as well as the expression of co-stimulatory molecules on the cell surface of antigen-presenting cells (macrophages, DCs, and B cells) (Tartey and Takeuchi, 2017[204]). Hence, this TLR-signaling plays a crucial role in the pathogenesis of skin inflammation and inflammatory diseases via generating different pro-inflammatory cytokines and other potential inflammatory mediators. The extracellular release of TNF-α exerts a direct cytotoxic effect as well as receptor-mediated NF-κB activation causing further activation of pro-inflammatory genes. Hence, exploring TLRs expressed on different skin cells and their activation during skin-inflammatory diseases will play a crucial role in understanding their immunopathogenesis. This will also help in repurposing different TLR agonists and antagonists in skin-inflammatory diseases, along with designing other novel molecules with high efficacy and low or negligible toxicity. 

## Future Perspectives and Conclusion

Skin-inflammatory diseases are on the rise, and they need specific attention in terms of identifying novel target molecules impacting their immunopathogenesis, having a great therapeutic potential to decrease the associated financial burden and social stigma. The discovery of TLRs has revolutionized the field of immunology, microbial pathogenesis, wound healing, along with developmental biology. Their discovery and further applications in different diseases have also led to the development of novel therapeutics and vaccine-based immunotherapeutics for several infections, inflammatory diseases, and cancers. The author has discussed the details of TLRs-based therapeutics for different inflammatory and infectious diseases somewhere else (Kumar, 2018[101]). Also, different host-derived endogenous negative regulators of TLR signaling have been described that have a potential for gene-based therapeutics to modulate TLR signaling (Kumar, 2020[102]). Thus, depending on the skin-inflammatory conditions, these endogenous negative regulators of TLR signaling, along with TLR agonists and antagonists, may have great potential in these skin-inflammatory diseases. However, pharmacokinetics and pharmacodynamics studies through different routes should be investigated. Although, the era of TLRs in skin-inflammatory diseases has just started and needs further studies in the direction. The expression of TLRs on dermal immune and non-immune cells have emerged as novel regulators of inflammatory skin diseases and their severity. Even these skin TLRs along with the TLRs in glial cells in the spinal cord, regulate the inflammation and neuroinflammation associated with chronic itch (pruritus) (Liu et al., 2012[118]; Taves and Ji, 2015[205]). Even sensory neurons express TLRs and directly sense the PAMPs and DAMPs may involve in pruritus that is an intractable symptom of different inflammatory skin diseases, including AD and psoriasis (Liu et al., 2012[118]; Trier et al., 2019[210]). Hence, TLRs expressed on skin cells and sensory neurons also control the feeling of pain and itch. 

In conclusion, TLRs are emerging knights in the field of skin inflammation and inflammatory diseases with a potential for therapeutic advancements. Future studies will open new TLR-based avenues to treat or prevent skin-inflammatory diseases.

## Conflict of interest

The author does not have any conflict of interest to disclose.

## Funding

The author has not received any funding from any funding source for this work.

## Contribution

The author has developed the idea, conceptualized, designed, and wrote the manuscript along with designing and drawing the figures and table.

## Figures and Tables

**Table 1:**
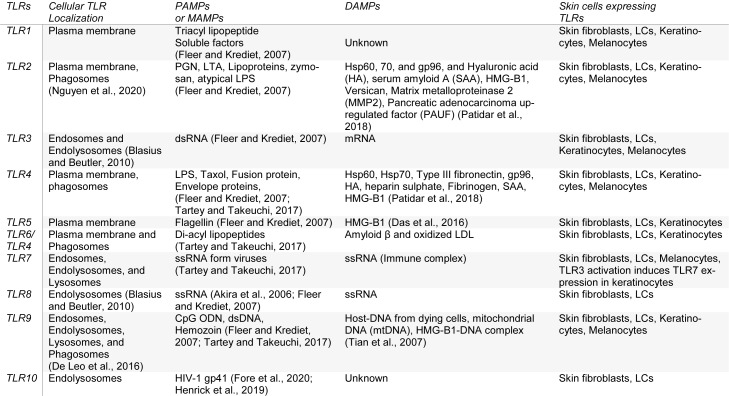
TLRs expressed in human skin cells, their location, PAMPs, and DAMPs
